# Intratracheal budesonide mixed with surfactant to increase survival free of bronchopulmonary dysplasia in extremely preterm infants: statistical analysis plan for the international, multicenter, randomized PLUSS trial

**DOI:** 10.1186/s13063-023-07650-0

**Published:** 2023-11-06

**Authors:** Kate L Francis, Christopher J D McKinlay, C Omar F Kamlin, Jeanie L Y Cheong, Peter A Dargaville, Jennifer A Dawson, Lex W Doyle, Susan E Jacobs, Peter G Davis, Susan M Donath, Brett J Manley

**Affiliations:** 1 Murdoch Children’s Research Institute, Melbourne, Australia; 2 Department of Paediatrics, The University of Melbourne, Melbourne, Australia; 3 Department of Paediatrics, Child and Youth Health, the University of Auckland, Kidz First Neonatal Care, Te Whatu Ora Counties Manukau, Auckland, New Zealand; 4 Newborn Research, The Royal Women’s Hospital, Melbourne, Australia; 5 Department of Obstetrics, Gynaecology and Newborn Health, The University of Melbourne, Melbourne, Australia; 6 The Royal Hobart Hospital, Hobart, Australia; 7 Menzies Institute for Medical Research, University of Tasmania, Hobart, Australia

## Abstract

**Background:**

Bronchopulmonary dysplasia (BPD), an inflammatory-mediated chronic lung disease, is common in extremely preterm infants born before 28 weeks’ gestation and is associated with an increased risk of adverse neurodevelopmental and respiratory outcomes in childhood. Effective and safe prophylactic therapies for BPD are urgently required. Systemic corticosteroids reduce rates of BPD in the short term but are associated with poorer neurodevelopmental outcomes if given to ventilated infants in the first week after birth. Intratracheal administration of corticosteroid admixed with exogenous surfactant could overcome these concerns by minimizing systemic sequelae. Several small, randomized trials have found intratracheal budesonide in a surfactant vehicle to be a promising therapy to increase survival free of BPD. The primary objective of the PLUSS trial is to determine whether intratracheal budesonide mixed with surfactant increases survival free of bronchopulmonary dysplasia (BPD) at 36 weeks’ postmenstrual age (PMA) in extremely preterm infants born before 28 weeks’ gestation.

**Methods:**

An international, multicenter, double-blinded, randomized trial of intratracheal budesonide (a corticosteroid) mixed with surfactant for extremely preterm infants to increase survival free of BPD at 36 weeks’ postmenstrual age (PMA; primary outcome). Extremely preterm infants aged < 48 h after birth are eligible if (1) they are mechanically ventilated, or (2) they are receiving non-invasive respiratory support and there is a clinical decision to treat with surfactant. The intervention is budesonide (0.25 mg/kg) mixed with poractant alfa (200 mg/kg first intervention, 100 mg/kg if second intervention), administered intratracheally via an endotracheal tube or thin catheter. The comparator is poractant alfa alone (at the same doses). Secondary outcomes include the components of the primary outcome (death, BPD prior to or at 36 weeks’ PMA), and potential systemic side effects of corticosteroids. Longer-term outcomes will be published separately, and include cost-effectiveness, early childhood health until 2 years of age, and neurodevelopmental outcomes at 2 years of age (corrected for prematurity).

**Statistical analysis plan:**

A sample size of 1038 infants (519 in each group) is required to provide 90% power to detect a relative increase in survival free of BPD of 20% (an absolute increase of 10%), from the anticipated event rate of 50% in the control arm to 60% in the intervention (budesonide) arm, alpha error 0.05. To allow for up to 2% of study withdrawals or losses to follow-up, PLUSS aimed to enroll a total of 1060 infants (530 in each arm). The binary primary outcome will be reported as the number and percentage of infants who were alive without BPD at 36 weeks’ PMA for each randomization group. To estimate the difference in risk (with 95% CI), between the treatment and control arms, binary regression (a generalized linear multivariable model with an identity link function and binomial distribution) will be used. Along with the primary outcome, the individual components of the primary outcome (death, and physiological BPD at 36 weeks’ PMA), will be reported by randomization group and, again, binary regression will be used to estimate the risk difference between the two treatment groups for survival and physiological BPD at 36 weeks’ PMA.

**Supplementary Information:**

The online version contains supplementary material available at 10.1186/s13063-023-07650-0.

## Study synopsis

The PLUSS trial is a multicenter, two-arm, parallel, double-blind, randomized controlled trial, enrolling at least 1060 extremely preterm infants in 21 participating hospitals across four countries (Australia, New Zealand, Canada, and Singapore). Enrolment commenced in January 2018 and was completed in March 2023. The trial is investigating whether intratracheal budesonide (a corticosteroid) combined with surfactant, compared with surfactant alone, will increase survival free of bronchopulmonary dysplasia (BPD) in extremely preterm infants.

Full details of the background to the trial and its design are presented in the published protocol [1].

### *Primary objective*

To determine whether intratracheal budesonide mixed with surfactant increases survival free of bronchopulmonary dysplasia (BPD) at 36 weeks’ postmenstrual age (PMA) in extremely preterm infants born before 28 weeks’ gestation.

### Secondary objectives

To determine whether intratracheal budesonide mixed with surfactant:

- ➢ Reduces BPD and/or BPD severity at 36 weeks’ PMA
- ➢ Reduces death before 36 weeks’ PMA
- ➢ Reduces death before hospital discharge
- ➢ Reduces other major neonatal morbidities (e.g., brain injury, retinopathy of prematurity, necrotizing enterocolitis, spontaneous intestinal perforation, pneumothorax requiring drainage, treated patent ductus arteriosus)
- ➢ Improves respiratory status at 40 weeks’ PMA
- ➢ Reduces the duration of hospitalization, respiratory support, and supplemental oxygen therapy

### Study population

Extremely preterm infants (specifically 22–27 weeks’ completed gestation) admitted to a participating neonatal intensive care unit (NICU), who fulfill the entry criteria detailed below.

#### Inclusion criteria (all must be satisfied)

1. Born before 28 weeks’ gestation
2. Less than 48 h of age
3. No more than one prior dose of exogenous surfactant administered
4. Receiving either:
  - Mechanical ventilation via an endotracheal tube, regardless of ventilation settings or oxygen requirement (automatically qualify for the intervention), or
  - Non-invasive respiratory support (any type including continuous positive airway pressure, nasal intermittent positive pressure ventilation [NIPPV], or nasal high flow) and there is a clinical decision to treat with surfactant (first or second dose)
5. Prospective, written, informed parental/guardian consent obtained

#### Exclusion criteria (any one or more mandates exclusion)

1. More than one prior surfactant dose
2. Prior treatment with postnatal corticosteroids for the prevention of lung disease (inhaled, nebulized, intratracheal, or systemic)
3. The infant is considered unlikely to survive the immediate postnatal transition and/or is not going to be admitted to the NICU
4. Known or suspected major congenital anomaly that is likely to affect respiratory status, including a postnatal clinical diagnosis of severe pulmonary hypoplasia following premature prolonged rupture of fetal membranes with resultant severe oligo- or anhydramnios, where the clinician feels survival is unlikely
5. The infant is likely to be transferred to a non-participating NICU within 24 h of birth

### Intervention

The intervention is budesonide (0.25 mg/kg) mixed with *poractant alfa* (200 mg/kg first intervention, 100 mg/kg if second intervention), administered intratracheally via an endotracheal tube or thin catheter.

### Randomization and blinding

The randomization schedule is provided by the Clinical Epidemiology and Biostatistics Unit at the Murdoch Children’s Research Institute, Melbourne, Australia. Randomization with balanced variable block sizes is used, stratified by study center, gestational age (22–25 weeks’ vs. 26–27 weeks’ completed gestation), prior surfactant therapy, and mode of respiratory support at randomization (mechanical ventilation via an endotracheal tube vs. non-invasive respiratory support).

When eligibility of an infant is confirmed, and prospective consent obtained, the infant is assigned to either receive surfactant plus budesonide, or surfactant alone, using a web-based randomization system with an allocation ratio of 1:1. A checklist on the website is used to confirm eligibility prior to randomization. Multiple births where more than one infant is eligible are randomized individually. A sealed opaque envelope at the study site is identified by the unique study ID generated from the web-based server (https://redcap.mcri.edu.au) [2, 3]. The sealed envelope is opened by a dedicated intervention team who are not providing direct clinical care to the infant and will not be involved in any future outcome assessments. Inside the main envelope are a further two sealed envelopes for the first and second interventions respectively. Infants remain in their allocated group for repeat interventions (if applicable), with each envelope remaining sealed until the intervention team is ready to prepare the allocated medication.

Parents/caregivers, direct healthcare providers, outcome assessors, data analysts, and trial investigators are blinded to the randomization group. Our experience is that it is virtually impossible to distinguish between the control and intervention study drugs, although there theoretically may be a subtle difference in the appearance of the surfactant with budesonide admixed. Additionally, the volume of the study drug to be administered is 0.5 mL/kg greater in the active treatment arm. To maintain blinding, the study drugs will be prepared by an independent intervention team whose members are not directly involved in the clinical care of the infant, and not involved in data collection or outcome assessments for the study. Data on the dose and type of intervention, as well as other data required by hospital pharmacies, will be recorded by the intervention team on allocation cards and stored in a secure lockbox only accessible by hospital pharmacists. In addition, after preparation of the intervention, the contents of the syringe will be covered using a stick-on label to obscure the volume and appearance to the bedside clinical staff. The pharmacy departments of each participating center and the Clinical Epidemiology and Biostatistics Unit will be the only other personnel aware of the allocated study intervention; they also will not be involved in data collection or outcome assessments for the study. Pharmacies will maintain a logbook of allocated study drugs and doses.

Neither the PLUSS Trial Steering Committee nor site researchers will be aware of the allocated interventions and will not be permitted access to this information until trial completion.

### Sample size

The estimated incidence of the composite primary outcome of survival free of BPD is 50%, based on a review of data from the lead center (The Royal Women’s Hospital, Melbourne, Australia) and published studies enrolling extremely preterm infants. With a sample size of 1038 infants (519 in each group), the study has 90% power to detect a relative increase in survival free of BPD of 20% (an absolute increase of 10%), from the anticipated event rate of 50% in the control arm to 60% in the intervention (budesonide) arm, alpha error 0.05. To allow for up to 2% study withdrawals or losses to follow-up, PLUSS aimed to enroll a total of 1060 infants (530 in each arm).

### Study procedures

Full details of the study procedures are presented in the published Study Protocol [1].

The intervention will be performed in participating tertiary NICUs with the medications prepared in the NICU (or potentially in the delivery room in some centers) after the birth weight of the infant has been confirmed. Following randomization, the first intervention will be administered as soon as possible. If the infant meets the same treatment criteria 6–12 h after the first intervention, a second (and final) intervention will be administered in the NICU.

In this pragmatic study, the following methods of intratracheal instillation will be permitted: standard bolus administration through an endotracheal tube that will remain in situ with ongoing mechanical ventilation, INSURE (INtubate, SURfactant, Extubate) technique via an endotracheal tube, or via a thin catheter in those infants receiving non-invasive respiratory support (including CPAP, NIPPV or nasal high-flow).

Dosing is as follows:

- *Poractant alfa* (both arms): 200 mg/kg initial dose; subsequent dose 100 mg/kg (if applicable)
- Budesonide (intervention arm only): 0.25 mg/kg (0.5 mL/kg of 1 mg/2 mL solution) added to each dose of *poractant alfa*.

## General statistical methodology 

### Objectives of analysis plan

This statistical analysis plan (SAP) is designed to cover the analysis of:

- The primary outcome
- In hospital secondary outcomes
- Outcomes related to the safety of the intervention

This SAP *does not* cover planned analysis of:

- Outcomes at 2 years of age (corrected for prematurity)
- Health economic evaluation
- Substudies

### Analysis software

Data will be exported from the study database to Stata (StataCorp 2019. Stata Statistical Software: Release 18.0 College Station, TX: StataCorp LLC) for analysis.

### Data verification

All data will be checked and cleaned by the trial data manager and trial statistician prior to analysis. Data analysis will not commence until the database is locked and the SAP has been submitted for publication.

### Definition of analysis populations

The analysis population will comprise at least 1038 infants (as per the sample size calculation) randomized to either receive the intervention (budesonide mixed with surfactant) or control (surfactant alone).

The intention-to-treat (ITT) population will include all randomized infants, regardless of exposure to the allocated treatment or adherence to the trial protocol, excluding infants who have been withdrawn from the trial. If a *per-protocol* analysis is performed (if requested by an editor or reviewer) it will exclude those infants who never received a trial intervention or received the wrong treatment (that is the treatment for the opposing arm to what they were allocated).

### Definitions of adverse events (AEs) and serious adverse events (SAEs)

AEs and SAEs are assessed from randomization until death, primary hospital discharge, or 52 weeks’ PMA, whichever occurs sooner. AEs are:

- Spontaneous intestinal perforation (perforation not associated with necrotizing enterocolitis or other known pathology)*
- The need for cardiopulmonary resuscitation (chest compressions) and/or administration of adrenaline/epinephrine (for resuscitation) within 24 h of the intervention*
- Pneumothorax requiring drainage (needle thoracocentesis or intercostal catheter insertion)
- Gastrointestinal hemorrhage, defined as fresh blood aspirated from an indwelling gastric tube, during the 14 days after the first intervention
- Clinical diagnosis of pulmonary hemorrhage within the first 48 h after the first intervention
- Any prescribed anti-hypertensive agents during the 14 days after the first intervention
- Hyperglycemia > 10 mmol/L and/or receiving insulin therapy during the 14 days after the first intervention
- Late-onset sepsis after 48 h of age, defined as a positive bacterial or fungal culture from a normally sterile site, or negative blood culture but clinical suspicion of sepsis and treatment with antibiotic/antifungal medication for ≥ 5 days)
- Oral candidiasis during the first 14 days after the first intervention
  *Defined as serious adverse events (SAEs)

In addition to the two SAEs above, all deaths before hospital discharge are defined as an SAE and are reviewed by the independent Data Safety Monitoring Board (DSMB) for relationship to the trial intervention and classified as a “respiratory death” or not.

### Adjustment for multiplicity

There will be no adjustment for multiplicity.

### Interim analyses

The DSMB have conducted multiple interim analyses for safety through the trial, and a single analysis for efficacy at the halfway point of recruitment when primary outcome endpoint data (death or BPD at 36 weeks’ PMA) were available for 530 infants (50% of the planned sample size). As per the PLUSS Trial DSMB Charter (version 2, 2019), “the DSMB may make a recommendation to cease the trial only in the presence of very strong (*P* < 0.001) interim evidence of a difference between groups in the primary outcome.” There is no adjustment for this interim analysis. The DSMB reviewed safety outcomes after the primary outcome was known for 50, 100, 265 (25% planned recruitment), 530 (50%), and 800 (75%) infants. At all time-points the DSMB recommended that the trial continue without changes to the protocol.

### Handling of missing data

The primary analysis will be based on an intention to treat population accounting for all infants randomized. If necessary, multiple imputation methods will be used for missing data; however, it is expected there will be very few instances in which the primary outcome cannot be determined. The reason for this is that infant death is always recorded and BPD assessment at 36 weeks will likely occur for nearly all infants when they are still in the hospital and their outcome can be recorded directly into the trial database. Therefore, we do not anticipate needing to address missing data for the primary or secondary outcomes in this study.

## Descriptive statistics

### Recruitment and follow-up

**Fig. 1:**
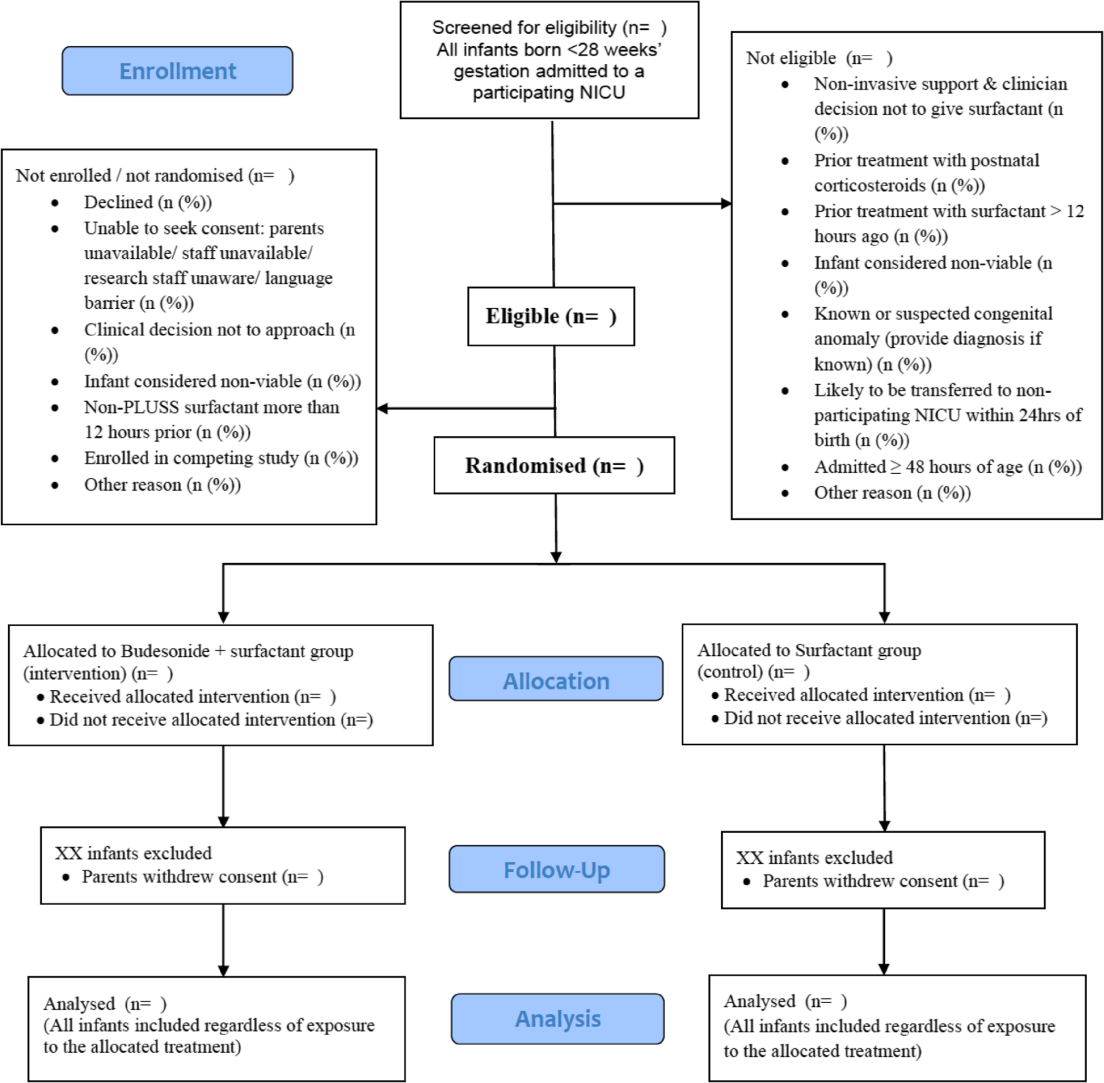
Consolidated Standards of Reporting Trials (CONSORT) 2010 flow diagram

All infants at participating hospitals were screened for eligibility for the trial. The CONSORT flow diagram (Fig. 1) will be used to report enrolment, randomization, intervention allocation, follow-up, and analysis groups.

### Baseline characteristics

Baseline characteristics will be summarized by group as shown in Example Table 1.

**Table 1:** Baseline characteristics

	Budesonide + surfactant group (*n* = XXX)	Surfactant group (control) (*n* = XXX)
Mothers:		
Age at delivery (years)	Mean (SD)	Mean (SD)
Ethnicity		
Aboriginal or Torres Strait Islander	*n* (%)	*n* (%)
Māori	*n* (%)	*n* (%)
Pasifika	*n* (%)	*n* (%)
First Nations, Ink/Inuit and/or Metis (Canada)	*n* (%)	*n* (%)
Asian	*n* (%)	*n* (%)
European (Caucasian)	*n* (%)	*n* (%)
Other	*n* (%)	*n* (%)
Exposure to any antenatal corticosteroids (yes)	*n* (%)	*n* (%)
Treatment with magnesium sulfate (yes)	*n* (%)	*n* (%)
Chorioamnionitis (clinical and/or histological) (yes)	*n* (%)	*n* (%)
Hypertensive disorders of pregnancy (yes)	*n* (%)	*n* (%)
Prolonged rupture of membranes > 18 h,	*n* (%)	*n* (%)
Labor (yes)	*n* (%)	*n* (%)
Cesarean section (yes)	*n* (%)	*n* (%)
Infants:		
Gestational age (weeks):	Mean (SD)	Mean (SD)
Birth weight (grams):	Mean (SD)	Mean (SD)
Small for gestational age (birth weight < 10th centile for gestation and sex)	*n* (%)	*n* (%)
Age at randomization (hours)	Mean (SD)	Mean (SD)
Male (yes)	*n* (%)	*n* (%)
Multiple birth (yes)	*n* (%)	*n* (%)
Intubated in the delivery room (yes)	*n* (%)	*n* (%)
Apgar score at 5 min	Median (IQR)	Median (IQR)
Clinical status before the first intervention		
Mechanically ventilated via an endotracheal tube (yes)	*n* (%)	*n* (%)
Surfactant treatment (yes)	*n* (%)	*n* (%)
Caffeine treatment (yes)	*n* (%)	*n* (%)
Inotrope treatment (yes)	*n* (%)	*n* (%)
Corticosteroids for hypotension (yes)	*n* (%)	*n* (%)
Blood gas analysis		
pH	Mean (SD) or median (IQR)	Mean (SD) or median (IQR)
pCO_2_ (kPa)		
Blood glucose concentration (mmol/L)		
Fraction of inspired oxygen immediately prior to first intervention	Mean (SD) or median (IQR)	Mean (SD) or median (IQR)

### Protocol deviations

Protocol deviations will not result in the exclusion of participants and will not be reported in the manuscript unless requested by editors or reviewers. We do not plan to perform a *per-protocol* analysis unless requested by editors or reviewers.

## Analysis of the primary outcome(s)

### Main analysis

The primary outcome is survival without physiological BPD at 36 weeks’ PMA.

Physiological BPD will be assessed between 36^+0^ and 36^+6^ weeks’ PMA, and infants will be defined as having BPD if any of the following criteria are met:

1. Receiving mechanical ventilation via an endotracheal tube, CPAP, NIPPV, or nasal high-flow ≥ 2 L/min, regardless of FiO_2_
2. An effective FiO_2_ ≥ 0.30, if receiving supplemental ambient oxygen or nasal prong flow < 2 L/min to maintain target oxygen saturations
3. An effective FiO_2_ < 0.30 if receiving supplemental ambient oxygen or nasal prong flow at < 2 L/min to maintain target oxygen saturations AND an unsuccessful air reduction trial.

For infants receiving oxygen by nasal prongs at < 2L/min, the effective FiO_2_will be determined using the Benaron-Benitz formula [4].

Any infants who are discharged home prior to 36 + 0 weeks’ PMA without any respiratory support or supplemental oxygen will be classified as “no BPD.”

The Trial Steering Committee has approved an algorithm for determining a diagnosis of BPD in cases where the BPD assessment is incorrectly or inadequately performed (e.g., too early, too late, or incomplete data). This algorithm uses data collected at exactly 36^+0^ weeks’ PMA and is assessed blinded to the treatment group. When this algorithm is required, infants will be defined as having BPD if any of the following criteria are met at 36^+0^ weeks’ PMA:

1. Receiving mechanical ventilation via an endotracheal tube, CPAP, NIPPV, or nasal high-flow ≥ 2 L/min
2. An effective FiO_2_≥ 0.22 if receiving supplemental ambient oxygen or nasal prong flow < 2 L/min to maintain target oxygen saturations, a conservative approach based on shared data from a recent large, randomized trial [5].

If a diagnosis of BPD is unable to be determined by this method, the Trial Steering Committee will assign a BPD status based on all available clinical evidence, blinded to the treatment group.

We will provide a table in the Supplementary Appendix of the number of infants in whom the BPD algorithm was used to determine BPD status and why the TSC was required to make a decision on the diagnosis of BPD, the number who required the effective FiO_2_ to be calculated to determine BPD, and the number who were discharged home prior to 36 + 0 weeks’ PMA without any respiratory support or supplemental oxygen (see Table S1).

The binary primary outcome will be reported as the number and percentage of infants who were alive without BPD at 36 weeks’ PMA for each randomization group. To estimate the difference in risk (with 95% CI), between the treatment and control arms we will use binary regression (a generalized linear multivariable model with an identity link function and binomial distribution). In the regression, the primary outcome will be the dependent variable, group allocation (intervention/control) is the predictor variable, and the stratification factors used in the randomization are covariates. Standard errors will be adjusted to take into account the clustering of multiple births.

The three randomization strata that will be reported by the treatment group in the primary outcome table and will be covariates in the adjusted binary regression model are:

1. Gestational age: 22–25 completed weeks’/26–27 completed weeks’
2. Prior surfactant therapy: no/yes
3. Mode of respiratory support at randomization: mechanical ventilation via an endotracheal tube/non-invasive respiratory support

Along with the primary outcome, the individual components of the primary outcome—death or physiological BPD at 36 weeks’ PMA, will be reported by a randomization group (*n* (%)). Again, binary regression as defined above will be used to estimate the risk difference between the two treatment groups for survival and physiological BPD at 36 weeks’ PMA (Example Table 2).

**Table 2:** Primary outcome and its components

Intention-to-treat analysis	Budesonide + surfactant group (*n* = XXX)	Surfactant group (*n* = XXX)	Adjusted risk difference (95% CI)^a^
Primary outcome			
Survival free of BPD at 36 weeks’ PMA	XX (%)	XX (%)	
Components of the primary outcome			
Alive at 36 + 0 weeks PMA	XX (%)	XX (%)	
BPD	XX (%)	XX (%)	

^a^Adjusted for stratification variables (gestational age, prior surfactant therapy, mode of respiratory support at randomization)

Additional information about deaths will appear in the Supplemental Appendix by treatment group, including a summary of the age at death, causes of death (categorized), what proportion of deaths were classed as “respiratory” by the DSMB, and the modes of death (see Table S2).

### Estimand for the PLUSS primary outcome

The addendum to the International Council for Harmonisation of Technical Requirements for Pharmaceuticals for Human Use (ICH) E9 (R1) guidelines [6] promotes the use of the estimand framework. An estimand consists of five attributes: population, treatment, variable of interest, i.e., outcome, summary measure, and possible intercurrent events (which is a post randomization event that can occur and preclude or affect the interpretation of the variable of interest, e.g., discontinuation of treatment). The primary outcome for the PLUSS trial is presented in the estimand framework below.

Primary outcome for the PLUSS trial presented in the estimand framework (table layout based on the proposed estimand framework reporting proposed by Kang et al. [7]).

**Table:** 

**Objective:** To determine the effect of intratracheal budesonide during the early treatment of respiratory distress syndrome (RDS) in extremely preterm infants increase?	
**Estimand:** The risk difference in survival without bronchopulmonary dysplasia (BPD) at 36 weeks’ postmenstrual age (PMA) in extremely preterm infants (22–27 weeks’ completed gestation) between those who received intratracheal budesonide plus surfactant with those who received surfactant alone	
**Treatment:** budesonide 0.25 mg/kg mixed with surfactant	
**Estimand**	**Analysis**
**Target population**	**Analysis set**
Extremely preterm infants 22–27 weeks’ completed gestation that are < 48 h of age, receiving mechanical ventilation via an endotracheal tube; OR infants receiving non-invasive respiratory support including CPAP, non-invasive intermittent positive pressure ventilation, or nasal high flow, and there is a clinical decision to treat the infant with exogenous surfactant	All randomized participants. Participants randomized to budesonide plus surfactant will be the active treatment group and those randomized to surfactant alone will be the comparator group
**Variable**	**Outcome measure**
Survival free of BPD at 36 weeks’ PMA	Death, or physiological BPD if any of the following criteria are met: 1. Receiving mechanical ventilation via an endotracheal tube, CPAP, NIPPV, or nasal high-flow ≥ 2 L/min, regardless of FiO_2_ 2. An effective FiO_2_ ≥ 0.30, if receiving supplemental ambient oxygen or nasal prong flow < 2 L/min to maintain target oxygen saturations 3. An effective FiO_2_ < 0.30 if receiving supplemental ambient oxygen or nasal prong flow at < 2 L/min to maintain target oxygen saturations AND an unsuccessful air reduction trial For infants receiving oxygen by nasal prongs at < 2 L/min, the effective FiO_2_will be determined using the Benaron-Benitz formula [4]. Any infants who are discharged home prior to 36 + 0 weeks’ PMA without any respiratory support or supplemental oxygen will be classified as “no BPD.”
**Handling of intercurrent events**	**Handling of missing data**
In the current study death is an intercurrent event that is included in the outcome variable (composite variable strategy)	In the current study missing data is anticipated to be very rare. Imputation is not planned
**Population-level summary measure**	**Analysis approach**
Risk difference between budesonide plus surfactant and surfactant alone	Adjusted risk difference (with 95% CI), between the treatment and control arms using binary regression (a generalized linear multivariable model with an identity link function and binomial distribution). The adjustment factors will be the stratification factors used in the randomization. Standard errors will be adjusted to take into account the clustering of multiple births

### Sensitivity analyses

If an imbalance in demographics from Table 1 is detected, a sensitivity analysis adjusting for the relevant demographics will be conducted for the primary outcome and its components.

A sensitivity analysis will be run for the primary outcome, excluding those infants who had their BPD status (yes/no) determined by using the BPD algorithm, and reported if an important effect on the primary outcome is seen.

### *Supplementary analyses*

We plan to include a Kaplan–Meier curve of survival up to hospital discharge or 52 weeks’ PMA (whichever comes first). If requested by journal editors or reviewers, we may perform other analyses that are not mentioned in this protocol. These will be performed consistently with the principles of this analysis plan, as far as possible.

#### Subgroup analyses

We acknowledge that the trial is not powered for subgroup analyses and therefore the analyses listed in this section are considered exploratory.

For the primary outcome subgroup analysis will be performed according to the pre-randomization strata: gestational age, exposure to surfactant prior to randomization, and mode of respiratory support at randomization. In addition, we plan to assess the effect of important factors that might modulate the risk of death and BPD, including sex, small for gestational age, and the presence of chorioamnionitis (Table 3).

**Table 3:** Results of the subgroup analysis

Primary outcome according to prespecified subgroup analyses	Budesonide + surfactant group (*n* = XXX)	Surfactant group (*n* = XXX)	Adjusted risk difference (95% CI)
**Randomization strata**			
Gestational age — no./total no. (%)			
22–25 weeks’ completed gestation	XX/XX(%)	XX/XX(%)	
26–27 weeks’ completed gestation	XX/XX(%)	XX/XX(%)	
Prior surfactant therapy — no./total no. (%)			
No	XX/XX(%)	XX/XX(%)	
Yes	XX/XX(%)	XX/XX(%)	
Mode of respiratory support at randomization — no./total no. (%)			
Mechanical ventilation via an endotracheal tube	XX/XX(%)	XX/XX(%)	
Non-invasive respiratory support	XX/XX(%)	XX/XX(%)	
**Factors that might modulate the effect of budesonide on the outcome of death and BPD**			Adjusted risk difference (95% CI)
Sex			
Male	XX/XX(%)	XX/XX(%)	
Female	XX/XX(%)	XX/XX(%)	
Small for gestational age (birth weight < 10^th^ centile for gestation and sex)			
No	XX/XX(%)	XX/XX(%)	
Yes	XX/XX(%)	XX/XX(%)	
Chorioamnionitis (clinical and/or histological diagnosis)			
No	XX/XX(%)	XX/XX(%)	
Yes	XX/XX(%)	XX/XX(%)	

**Fig. 2:**
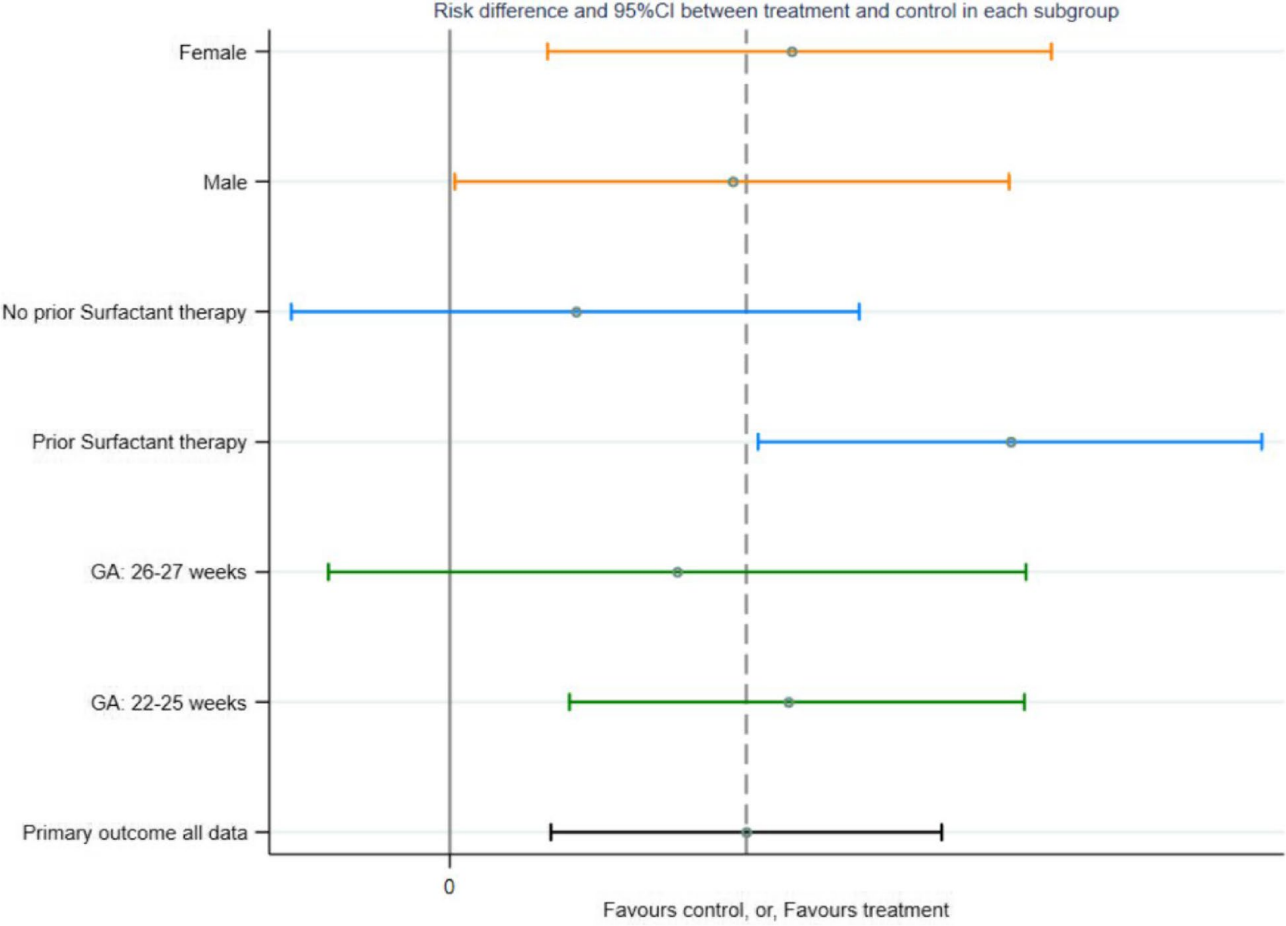
Plot of the individual subgroup risk differences (95% CI) for the primary outcome

The first step in the subgroup analysis will be to run a binomial regression (with stratification variables as covariates) with an interaction term between the subgroup variable and the treatment arm (Example Table 3). If there is no evidence of interaction (*p* > 0.05), any differences between subgroups will be regarded as due to chance. Where there is evidence of an interaction, the adjusted risk difference (95% CI) between the treatment and control arms will be reported for the subgroup (Example Table 5). A plot will also be created that will include the primary outcome estimate (95% CI) (Example Fig. 2) and each subgroup estimate (95% CI) (Fig. 1).

## Secondary outcomes

### Main analysis

The secondary outcomes will be split into two tables “in hospital secondary outcomes” and “adverse events.” Binary secondary outcomes will be presented as the number and percentage of infants, by randomization group. The risk difference (with 95% CI) between the treatment and control arms will be estimated by binary regression (a generalized linear multivariable model with an identity link function and binomial distribution).

Continuous secondary outcomes will be graphed and visually inspected to determine if the distribution of values is skewed. If there is no indication that the distribution is skewed the mean and standard deviation (SD) will be reported for the variable, by randomization group. Linear regression will be used to estimate the difference of means (with 95% CI) between the treatment and control groups. For continuous variables where the data are judged to be skewed the median and interquartile range (IQR) will be reported, by randomization group. Quantile regression will be used to estimate the difference of medians (with 95% CI) between the treatment and control arms for these variables.

We will report the number of interventions received by both groups in the text.

There will be no adjustment for stratification variables for the secondary outcomes. Standard errors will be adjusted to take into account the clustering of multiple births. The analysis will be performed on the intention-to-treat population. There will be no adjustment for multiplicity. The results of these analyses will be presented in Example Tables 4 and 5.

**Table 4:** In hospital secondary outcomes. Note: some included definitions will appear in the text in the manuscript or in table footnotes

	Budesonide + surfactant group (*n* = XXX)	Surfactant group (*n* = XXX)	Risk/mean/median difference (95% CI)
BPD severity/grade at 36 weeks’ PMA [8]:			
Mild (grade 1)	*n*/*n* (%)	*n*/*n* (%)	
Moderate (grade 2)	*n*/*n* (%)	*n*/*n* (%)	
Severe (grade 3)	*n*/*n* (%)	*n*/*n* (%)	
Mode of respiratory support received at time of BPD assessment:			
Mechanical ventilation via an endotracheal tube	*n*/*n* (%)	*n*/*n* (%)	
CPAP or NIPPV	*n*/*n* (%)	*n*/*n* (%)	
Nasal high-flow >= 2 L/min	*n*/*n* (%)	*n*/*n* (%)	
Supplemental oxygen only	*n*/*n* (%)	*n*/*n* (%)	
None	*n*/*n* (%)	*n*/*n* (%)	
Clinical BPD at 40 + 0 weeks’ PMA, defined as receiving any supplemental oxygen or any form of respiratory support	*n*/*n* (%)	*n*/*n* (%)	
Treatment with postnatal systemic corticosteroids for lung disease	*n*/*n* (%)	*n*/*n* (%)	
Severe brain injury on cranial ultrasound: severe (grade III or IV) intraventricular hemorrhage, and/or cystic periventricular leukomalacia	*n*/*n* (%)	*n*/*n* (%)	
Severe (stage 2 or above) retinopathy of prematurity (ROP), and/or ROP treated with laser, cryotherapy, or intraocular therapy	*n*/*n* (%)	*n*/*n* (%)	
Necrotizing enterocolitis, modified Bell’s criteria stage 2 or greater	*n*/*n* (%)	*n*/*n* (%)	
Patent ductus arteriosus treated with anti-prostaglandin therapy or surgical ligation	*n*/*n* (%)	*n*/*n* (%)	
Total duration of mechanical ventilation via an endotracheal tube (days)	median (IQR)	median (IQR)	
Discharged home on oxygen or receiving supplemental oxygen in hospital beyond 52 weeks’ PMA	*n*/*n* (%)	*n*/*n* (%)	
PMA at cessation of positive pressure respiratory support (mechanical ventilation via an endotracheal tube, CPAP, NIPPV, nasal high-flow, or other positive pressure respiratory support)(weeks)	Mean (sd) or median (IQR)	Mean (sd) or median (IQR)	
PMA at cessation of supplemental oxygen (weeks)	Mean (sd) or median (IQR)	Mean (sd) or median (IQR)	
Length of hospital stay (days)	Mean (sd) or median (IQR)	Mean (sd) or median (IQR)	
Z-scores at 36 weeks’ PMA			
Weight	Mean (sd)	Mean (sd)	
Length	Mean (sd)	Mean (sd)	
Head circumference	Mean (sd)	Mean (sd)	
Body mass index	Mean (sd)	Mean (sd)

**Table 5:** Adverse events

	Budesonide + surfactant group (*n* = XXX)	Surfactant group (*n* = XXX)	Risk difference (95% CI)
^a^Spontaneous intestinal perforation	*n*/*n* (%)	*n*/*n* (%)	
^a^Cardiopulmonary resuscitation and/or epinephrine within 24 h of the intervention	*n*/*n* (%)	*n*/*n* (%)	
Pneumothorax requiring drainage	*n*/*n* (%)	*n*/*n* (%)	
Gastrointestinal hemorrhage < 14 days after the first intervention	*n*/*n* (%)	*n*/*n* (%)	
Pulmonary hemorrhage < 48 h after the first intervention	*n*/*n* (%)	*n*/*n* (%)	
Anti-hypertensive agents < 14 days after the first intervention	*n*/*n* (%)	*n*/*n* (%)	
Hyperglycemia > 10 mmol/L and/or receiving insulin therapy < 14 days after the first intervention	*n*/*n* (%)	*n*/*n* (%)	
Late onset sepsis	*n*/*n* (%)	*n*/*n* (%)	
Oral candidiasis < 14 days after the first intervention	*n*/*n* (%)	*n*/*n* (%)	

^a^Serious adverse events

Key respiratory parameters in the first 14 days after randomization (e.g., receiving mechanical ventilation via an endotracheal tube, FiO_2_) may be presented graphically. These figures may be included in the main paper or Supplementary Appendix.

## Safety outcomes

The adverse events listed in the “Definitions of adverse events (AEs) and serious adverse events (SAEs)” section are part of the secondary outcomes which are reported in Table 5. All the AE variables are binary variables and will be reported as number and percentage. The risk difference (95% CI) between the treatment and control groups will be estimated using binary regression models (a generalized linear multivariable model with an identity link function and binomial distribution), with the AE event being the dependent variable and the treatment group the predictor variable.

## Exploratory outcomes

If requested by journal editors or reviewers, we may perform other analyses that are not directly mentioned in this protocol. These will be performed consistently with the principles of this analysis plan, as far as possible.

Subsequent analyses of a more exploratory nature will not be bound by the strategy described in this SAP but are expected to follow the broad principles described. This subsequent work would have its own SAP and any results interpreted with consideration of the primary outcome as per this SAP.

### Supplementary Information

**Additional file 1.** **Table S1.** Example Table of reasons for using the “BPD algorithm” to diagnose BPD. **Table S2.** Example Table of additional death data to be reported.

**Additional file 2.** 

## Data Availability

Not applicable.

## Abbreviations

AE: Adverse event
BPD: Bronchopulmonary dysplasia
CI: Confidence interval
CPAP: Continuous positive airway pressure
DSMB: Data and Safety Monitoring Board
FiO_2_: Fraction of inspired oxygen
IQR: Interquartile range
NICU: Neonatal intensive care unit
NIPPV: Nasal intermittent positive pressure ventilation
pCO_2_: Partial pressure of carbon dioxide
PLUSS: The *P*reventing *L*ung Disease *U*sing *S*urfactant + *S*teroid Trial
PMA: Post-menstrual age
SAE: Serious adverse event
SAP: Statistical analysis plan
SD: Standard deviation
