# Supplementary material for: Intratracheal budesonide mixed with surfactant to increase survival free of bronchopulmonary dysplasia in extremely preterm infants: statistical analysis plan for the international, multicenter, randomized PLUSS trial
Source: Trials. 2023 Nov 6;24:709. doi: 10.1186/s13063-023-07650-0 (PMC10629198; doi:10.1186/s13063-023-07650-0)
Supplement: Supplementary file 1 — Additional file 1. Table S1. Example Table of reasons for using the “BPD algorithm” to diagnose BPD. Table S2. Example Table of additional death data to be reported. [file 13063_2023_7650_MOESM1_ESM.docx]

**Table S1. Example Table of reasons for using the “BPD algorithm” to diagnose BPD.**

|  | Number of infants |
| --- | --- |
| Wrong method used to determine BPD |  |
| BPD assessment not done |  |
| BPD assessment done early <36+0 weeks’ PMA |  |
| BPD assessment done late >36+6 weeks’ PMA |  |
| Discharged before 36+0 weeks PMA |  |
| Effective FiO_2_ used |  |
| Total number of BPD reviews: |  |

**Table S2. Example Table of additional death data to be reported.**

|  | Budesonide + surfactant group  (n = XXX) | Surfactant group  (n = XXX) |
| --- | --- | --- |
| Death before hospital discharge | n/n(%) | n/n(%) |
| Respiratory death (classified by DSMB) | n/n(%) | n/n(%) |
| Age of death, days | median (IQR) | median (IQR) |
| Causes of death (categorized)  1.  2.  … | n/n(%)  n/n(%)  n/n(%) | n/n(%)  n/n(%)  n/n(%) |
| Mode of death |  |  |
| During cardiopulmonary resuscitation (CPR) | n/n(%) | n/n(%) |
| Life support withdrawn in a moribund infant (not during CPR) | n/n(%) | n/n(%) |
| Life support withdrawn after consultation with parents (redirection of care due to concerns regarding quality of life) | n/n(%) | n/n(%) |
